# Exploring the experiences of dentistry students of Tabriz University of Medical Sciences of academic motivation: a content analysis study

**DOI:** 10.1186/s12909-024-05237-0

**Published:** 2024-03-06

**Authors:** Katayoun Katebi, Saeideh Ghaffarifar, Gholamali Dehghani, Ahmad Pourabbas

**Affiliations:** 1 Department of Medical Education, Faculty of Medicine, Tabriz University of Medical Sciences, Tabriz, Iran; 2 Department of Oral and Maxillofacial Medicine, Faculty of Dentistry, Tabriz University of Medical Sciences, Tabriz, Iran; 3 Medical Education Research Center, Health Management and Safety Promotion Research Institute, Tabriz University of Medical Sciences, Tabriz, Iran

**Keywords:** Motivation, Students, Dental, Qualitative research

## Abstract

**Background:**

Students’ academic motivation is crucial to their academic performance, success, and future career performance. Understanding the experiences of students regarding academic motivation can help address this issue. This qualitative study aimed to explore the experiences of dentistry students of Tabriz University of Medical Sciences regarding academic motivation.

**Methods:**

This qualitative content analysis study collected data through semi-structured individual interviews with open-ended questions. The collected data were then organized into codes, subcategories, categories, and themes. Extensive interviews, meetings, and active engagement with the participants were conducted to ensure the strength of the data collected.

**Results:**

The results of this study yielded 20 subclasses and 11 classes. These codes, categories, and sub-categories were summarized into seven themes: self-efficacy, outcome expectations, outcome expectancies, emotional coping, self-regulation, situational perception, and environment.

**Conclusion:**

This study showed that various concepts, from personal processes to environmental and social processes, play a role in students’ academic motivation. This study’s findings can contribute to developing more effective interventions to improve the motivation level of dentistry students.

## Introduction

Motivation, an internal force propelling an individual, plays a pivotal role in understanding behavior, predicting outcomes, and guiding actions toward goals [1]. It energizes individuals, directs activities, and contributes to behavioral development [2]. One crucial aspect impacting academic progress is motivation [3]. Academic motivation encompasses the joy of learning, directed mastery, curiosity, resilience, and engagement in challenging tasks [4].

Highly motivated students exhibit focused attention in class, produce high-quality assignments, explore learning beyond the classroom, and demonstrate increased problem-solving abilities [1, 5]. When learners have the necessary motivation to learn, the process of communication is facilitated, content becomes more fluent, anxiety is reduced, and creativity and learning are expressed [6], contributing to a positive educational experience for both students and professors [7].

Studies reveal that individuals with high academic motivation undertake more activities, complete assignments diligently, and achieve greater success [8]. However, disruptions in motivation stemming from a perceived lack of alignment between educational or professional activities and personal goals can lead to decreased motivation and hesitancy.

Motivational disruptions may manifest as pessimism, anxiety, and depression, impacting personal, social, and professional performance and ultimately influencing academic achievement negatively. Many interconnected factors, such as learner characteristics, curriculum quality, teacher attributes, educational environment, and learning tasks, influence learners’ interest and motivation [9].

Undergraduate dentistry training in Iran takes six years, and the graduates receive a Doctor of Dental Surgery degree. The main admission requirements are a high school diploma and acceptance in the nationwide official university entrance exam held once yearly. In this program, students pass basic sciences in two years and then enter the pre-clinic phase for about two years, followed by two years of the clinical phase, in which the students practice dentistry in clinical departments under the supervision of the faculty members. Dentistry is a very popular field, and only the students who score high in the highly competitive university entrance exam can enter it.

Since educational centers are always faced with learners who seem to lack motivation, understanding these factors is crucial for educators to choose effective teaching measures, enhance interaction, and ultimately increase teaching effectiveness [10, 11]. Factors affecting academic motivation include job guarantee, job identity, university facilities, educational level, and the learning environment [12, 13]. Qualitative research in Sweden on medical and nursing students identified the relevance of course topics to future job needs as a significant factor influencing academic motivation [14].

Despite some studies that have evaluated the factors that influence medical science students’ academic motivation, there has been limited research exploring the perspectives of dentistry students in Iran regarding the issue. Recognizing this gap, the primary objective of this qualitative content analysis study was to explore the experiences of dentistry students at Tabriz University of Medical Sciences in 2023 regarding their academic motivation.

## Methods

The content analysis approach was used for this qualitative research. This method contributes to deep exploration of experience and understanding of the data, leading to conclusions about the meaning of these experiences [15].

### Context

This study took place in Tabriz University of Medical Sciences, Tabriz, Iran, a first-grade university in Northwest Iran. The students attending the dentistry faculty of this university are among the top-grade students from the national university entry exam.

### Participants

The participants were selected by purposeful sampling from the dentistry students of Tabriz University of Medical Sciences by considering diversity in terms of age, gender, entry year, place of residence, and marital status. The students who were from Tabriz city and lived with their parents were considered native and any student not from Tabriz city was considered non-native. The international students were excluded. Purposeful sampling continued until no new class or topic connected with the desired concept emerged. Selected individuals received a phone call explaining research objectives and methods, inviting them for an interview held in the Department of Oral Medicine’s seminar room.

### Interviews

Data was collected through semi-structured one-on-one interviews with open-ended questions from dentistry students of Tabriz University of Medical Sciences [16]. Interviews with participants continued until the research team concluded that conducting further interviews would not add new data and that emerging themes were repetitive.

The aim of the study was explained to the participants, but to avoid bias, the interviews began with a general open-ended question, “Please describe one day of your presence at the faculty in detail from the moment you enter the faculty until you leave?“. Moreover, to have a deeper insight into the subject, more detailed and follow-up questions were raised according to the participants’ responses, to maintain the conversation in line with the research aim. Probing questions included: “What do you mean by…?“, “Can you explain further about…?“, “Please provide an example?“, “How did you feel when that happened?” and “What caused you to make that decision?”. The interviewer tried to use fewer leading questions to avoid leading the answers.

All interviews were recorded with the participant’s permission, and the purpose of recording the conversations was explained. In addition to digital recordings, observation methods, and field notes were used to capture the participants’ tone of voice, body posture, and gestures. Immediately after each interview, the text was analyzed, and the unit of analysis was selected. Open codes were then extracted manually by two independent researchers. This process guided subsequent interviews and determined the selection of the next interviewee.

### Data analysis

The interviews were analyzed using the content analysis method, following the steps suggested by Granheim and Lundman [17]. First, immediately after each interview, the text was transcribed word for word and reviewed multiple times by two independent researchers. The participants were given the opportunity to review and provide feedback on the transcripts, and further contact was made if necessary.

Each researcher then wrote an interpretive summary for each interview. These summaries were reviewed multiple times and broken down into smaller semantic units or codes. The primary codes were compared, and similar codes were grouped into subcategories [18]. Another researcher also reviewed and studied the initial coding process.

Through constant comparison, codes representing a single topic were grouped into sub-categories and categories. Ambiguous points that required attention were addressed by reviewing them with the participants and exploring them in subsequent interviews. This process ensured that any ambiguities were resolved and the placement of codes in each category was fully specified. The categories were then classified into themes. The results were then shared with a number of participants to compare and confirm the congruence of the emerging ideas with their own experiences.

Then, various motivational theories were studied and the compatibility of the categories obtained from this study with each of the theories was evaluated and finally it was determined that the concepts obtained from the present study are most compatible with the social cognitive theory.

### Rigor

Different strategies were implemented to ensure dependability, transferability, confirmability, and credibility throughout the research process [19]. For dependability, the interviews and the extracted codes and categories were sent to the research team members and other colleagues who were not involved in the research. For the purpose of auditing, all the research steps were described in detail so that the external observer could repeat the process. For transferability, various sources of information were used to collect data, including native, non-native, male, and female students, and a search was also made for negative and inconsistent items. The research environment was clearly described in order to clarify the context of the research. The explanation of the data was matched with several theories, and conformability ensued by returning the results to the participants to assess whether the findings reflected their opinions. The credibility was ensued by lengthy interaction and interviews with the participants. The coded text of the extracted data was returned to the participants for information confirmation through member checking. The results of this research were given to the people who did not participate to judge the findings according to their own experiences.

### Ethical considerations

The implementation of the project started after receiving approval from the ethics committee. The study’s objectives, the participants’ cooperation, and the method of data collection and recording were explained to the participants, and they were included in the study if they had informed consent and gave informed written consent. The participants were assured that all information, such as the names of the participants, interview files, and writings, were kept confidential, and they could leave the study whenever they wanted. Study ethics code is IR.TBZMED.REC.1401.494.

## Findings

Among the 16 participants in this research, nine were female, and seven were male. Their ages ranged from 20 to 27 years, and they were dentistry students in their second to sixth years of study. Seven students identified as native, while nine were non-native. Among nine non-natives, six participants resided in the dormitory, and three lived in student housing (Table 1).

**Table 1:** Characteristics of the participants (*N* = 16) from dentistry students of Tabriz University of Medical Sciences

Code	Gender	Age	Living status	Academic Year	Marital status
1	Female	22	Dormitory	4	Single
2	Female	25	Dormitory	4	Married
3	Female	22	Native	4	Single
4	Male	23	Native	4	Single
5	Male	22	Dormitory	5	Single
6	Male	27	Student Housing	6	Married
7	Male	25	Native	6	Single
8	Female	23	Native	5	Single
9	Male	20	Native	2	Single
10	Female	22	Dormitory	4	Married
11	Male	24	Native	6	Single
12	Female	27	Student Housing	5	Married
13	Male	26	Student Housing	6	Married
14	Female	21	Dormitory	2	Married
15	Female	22	Native	5	Single
16	Female	23	Dormitory	5	Single

Analysis of the interview data resulted in 202 primary codes based on 20 subcategories, and 11 categories were identified (Table 2).

**Table 2:** Examples of extracting codes, subcategories, and categories regarding experiences of dentistry students of Tabriz University of Medical Sciences of academic motivation using content analysis method

Meaning unit	Codes	Sub-category	Category
Camping and movie theater activities are missing from our program. The professors must encourage students to participate in research activities. Some patients don’t take us seriously. Holding prized competitions can help students to be more active. Office clerks only work when we have classes.	Extracurricular activities, Research encouragement, Inappropriate patient behavior towards students, Competitive student environment, Convenient operating hours for students	Institutional support and Motivational Atmosphere	Social Environment

In total, all these codes, subcategories, and categories were placed in 7 themes, which are summarized in Table 3:

**Table 3:** Subcategories, categories, and themes extracted from the interviews of dentistry students of Tabriz University of Medical Sciences of academic motivation using content analysis method

Sub-Categories	Categories	Theme
The experiences of the past semester	Self-evaluation	Self-efficacy
Discrepancy between Theoretical Learning and Practical Needs		
Experiences of Senior Students	Social comparison	
Future social recognition	Social outcome	Outcome expectations
Future Job positions	Physical outcome	
Future economic status		
Proficiency (Sense of accomplishment)	Social value	Outcome expectancies
Interest in the field		
Conscience		
Anxiety	Peace of mind	Emotional coping
Fear		
Well-being		
Planning for a heavy course load and work pressure (time management)	Goal setting	Self-control
Prioritizing recreation	Effort allocation	
Peer interactions		
Prioritizing high income from employment during education		
Prioritizing family matters		
Discontent with high student density	Adaptation	Situational perception
Cultural attributes	Social environment	Environment
Institutional support and motivational atmosphere		
Professor-centered challenges		
University service quality	Physical environment	

### Self-efficacy

The self-efficacy theme included two categories: self-evaluation and social comparison. Successful academic experiences enhance students’ interest in various courses, while unsuccessful experiences lead to disinterest. Self-evaluation, highlighted by students, reflects on personal accomplishments and setbacks. For instance, a 23-year-old female student shared her thoughts on failing the basic sciences exam and its impact on her academic journey:

*“Well, I failed the basic sciences exam. If I had passed the exam in the first year, I would be graduating next year, which bothers me a lot.”*

The lack of scientific adequacy in some courses, the mismatch between theoretical and practical content, and the need for further enrichment in the field indicate a gap between theoretical concepts and clinical work,” voiced a student, highlighting challenges in bridging theoretical knowledge with clinical applications:

*“For example, when we encounter a calcified tooth, while we have not been given any practical training on how to treat it, we get disinterested on the subject because we don’t know how to do it.”*

Comparing oneself with others significantly influences motivation. Observing the success or failure of peers and residents, shapes students’ perceptions. Disappointing conversations of others, especially newly graduated dentists or final year students, are often seen in students’ conversations:

*“I’ve heard from different dentists that they do not teach you anything at university. Well, what’s the point of me attending classes and spending all this time if I’m not learning?“*

expressed a student, reflecting on the discouraging sentiments of young dentists.

*“I see newly graduated specialists or residents, who worked so hard and were among the best, but now they have nothing, I come to the conclusion that it is not worth it to try so hard.”*

### Outcome expectations

The theme of outcome expectations comprised two categories: social outcomes and physical outcomes. Students consider social status when choosing a profession. The desire for a prestigious position, acts as a motivator for some students:

*“Now, one of the motivations that I have is that I want to be a faculty member.”*

Many students enter dentistry expecting higher income and better job prospects compared to other fields. The perception of dentistry as a lucrative field, combined with the shorter duration of study compared to medicine, attracts candidates.

*“An important issue is mainly the job market, which is very important. Most of us entered this field with the idea that it is medicine and we can help people, but more importantly the other side is the job market.”*

### Outcome expectancies

Interest in dental sciences and a general passion for dentistry emerge as critical concept on academic motivation. The sense of responsibility, sense of accomplishment, and the learning experience associated with patient care motivate students. Having a sense of responsibility towards patients and feeling the need to provide them with the highest quality dental services was another thing that came up. In this regard, a student said:

*“One of the best parts of our education is where we take the responsibility of a patient, the feeling of being a doctor, especially now that we are dealing directly with the patient.”*

Or another student said:

*“If you are not interested in your work, maybe you will continue for a while, but then you will be discouraged. The only thing left is what motivated you to choose this field”*.

emphasizing the importance of genuine interest in sustaining motivation.

### Emotional coping

Stress was one of the most frequent concepts in students’ conversations. This stress was not limited to certain situations such as exams and included many situations.

*“Stress means that we don’t enjoy what we are doing.”*

Or another student said

*“Instilling a sense of fear that can negatively impact performance, weaken one’s capabilities. Creating fear may hinder rather than empower an individual to excel in their work.”*

### Self-control

Goal setting and effort allocation are the two categories in self-control theme. A substantial number of courses can result in fatigue and burnout, leading to the manifestation of negative attitudes, behavior, and feelings under intense psychological pressure. This recurring theme emerged frequently in student discussions.

*“Our schedule is incredibly demanding, leaving us no time to study at home. Our only available time is on the weekends. The current workload is becoming overwhelming.”*

Another student emphasizes giving priority to fun enjoyment:

*“Before coming to the university each morning, I engage in activities like jogging, exercising, and having breakfast. Unfortunately, this routine often causes me to miss morning classes.”*

Economic challenges often compel students to work long hours to meet basic needs, but some prioritize work over studies for further well-being.

*“I couldn’t afford to solely focus on studying due to financial constraints. Living with family provides better conditions, but independence demands managing daily tasks alongside studying, work, and responsibilities. Balancing these demands becomes challenging, especially for those working part-time.”*

The desire to have fun, especially in environments where family control is limited, emerged as a significant concept among students. Concerns about negative influences from peers were raised by a student who noted that:

*“Most of my classmates who act irresponsibly are a group of friends.”*

Distance from the family, the responsibilities of managing the family, and the simultaneous education of the spouse in another city were some of the concepts that were raised in this category.

*" It is also important that the student’s mind is not occupied with other things. For example, when my family has a problem, I can’t attend classes with joy and learn.”*

### Situational perception

Classes with a large number of students, around 80, pose challenges for engagement. Smaller classes of 40 or 20 students allow better interaction between teachers and students, fostering a more focused learning environment. In clinical units, students expressed concerns about patient-student ratios, with some highlighting difficulties in meeting requirements.

*“We were required to treat eight patients in a course, but finding them is challenging. If there wasn’t so much admission of students, we could study more easily.”*

### Environment

The theme of environment included two categories: social and physical environment. The university’s motivating and supportive atmosphere encompasses extracurricular activities, research encouragement, inappropriate patient behavior toward students, and a competitive student environment. Establishing a conducive learning environment, crucial for cultivating highly qualified students, involves effective factors such as extracurricular activities.

*“There’s a lack of cultural exposure. Integrating cultural discussions, perhaps through documentaries, and organizing recreational activities like camping can contribute to a more holistic educational experience.”*

One of the students said:

### “There’s a decline in student involvement in research activities.”

Inappropriate and insulting patient behavior negatively impacts student motivation, highlighting challenges in clinical departments. For example, one of the students raised:

*“Instances of patients questioning students’ competence affect our morale, for example, by saying: is it your first time seeing a patient?”*

Challenges related to professors include inappropriate behavior, teaching quality, and deficiency in Exam Outcome Clarity.

According to the participants’ statements, professors who humiliate students affect their motivation. One of the contributors said

Insulting us for doing something wrong in front of patients really bothers me.

Issues such as the use of passive teaching methods and the lack of use of new educational technologies to make course materials attractive were raised by students.

Exams are considered an important part of education, and standard exams make students see the results of their efforts. if they succeed in the exam, they continue to study with more motivation, and if they fail in the exam, they try harder. If the tests are not standard, students may not attribute the reason for not succeeding in the test to their low effort.

*“Some exams are such that there is no difference between those who study and those who do not. But, well, the professors can grade in a way that makes a difference between those who study and those who don’t.”*

The quality of university services, including dormitory and educational facilities, was mentioned by many students. Several students mentioned the inappropriate condition of student dormitories.

*“The gym is near the library, and there is so much noise that I cannot concentrate on my studies.”*

Educational facilities were one of the most frequent items in students’ conversations. The students mentioned things such as the old equipment of the clinics and the lack of materials and tools for practical training.

*“The welfare situations they provide us are very limited.“*

## Discussion

This qualitative content analysis aimed to explore the experiences of dentistry students at Tabriz University of Medical Sciences in 2023 regarding their academic motivation. The study findings revealed that academic motivation encompasses seven themes: self-efficacy, outcome expectations, outcome expectancies, emotional coping, self-regulation, situational perception, and environment. Motivation for academic success reflects an individual’s inclination to overcome challenges, strive for excellence, uphold high standards, and exhibit a drive to advance and achieve personal and professional goals [20]. Individuals with high motivation to progress display a persistent focus on success, exhibit confidence in their decisions, engage in prolonged concentration on tasks, demonstrate resilience in the face of obstacles, and intensify efforts to avert failure [21].

### Self-efficacy

The self-efficacy theme included two categories of self-evaluation and social comparison. Believing that learners are making progress can create self-efficacy. As learners assess their progress toward goals, any perceived discrepancy between the set goal and actual progress can prompt increased effort and persistence. The belief that progress is being made plays a pivotal role in fostering self-efficacy [22].

A prevalent concept in this research was the gap between learning and job requirements. The lack of academic adequacy in certain practical sections stems from potential discrepancies between theoretical and practical courses. Course design-related issues, such as insufficient practical units, contribute to students’ motivational challenges. Addressing the professional needs of dental graduates early in the course requires adjustments to educational programs for a more skilled and productive workforce. Ravipour et al. identified career future, professional identity, and appropriateness of educational courses as pivotal factors influencing the academic motivation of environmental health engineering students [23], aligning with the present study’s findings.

In the context of evidence theory, individuals’ perceptions of the causes behind their successes and failures profoundly shape their motivation. This implies that responses to events hinge on happiness in success and sadness in failure [24].

Social comparison emerges as a significant concept in this study, with students comparing themselves to residents and recently graduated dentists. Social comparison posits that such comparisons influence attitudes and behaviors—favorable comparisons motivate, while unfavorable ones lead to diminished effort [25].

### Outcome expectations

Pursuing a high income through obtaining a dental degree emerged as a significant aspect. This finding aligns with Dikic et al.‘s study on medical students in Turkey, where financial gain and improved job positions were highlighted as crucial factors influencing students’ field choices [26]. Nejat et al.‘s study emphasized the significance of social prestige and the medical field’s importance in society as reasons for choosing the medical profession [27]. Similarly, Nemat Elahi’s study at the Faculty of Dentistry of Mashhad University of Medical Sciences identified high job positions, substantial income, self-employment potential, and the artistic nature of the field as reasons for high motivation in the participants [28]. These studies support the present research’s findings regarding future social status and economic factors.

While external motivations, such as financial incentives, play a crucial role in education, relying solely on these factors may yield undesirable consequences. If the primary goal for medical science graduates is solely better job positions and increased income, it could impede their ability to contribute to society effectively through their services.

### Outcome expectancies

Throughout this study, students highlighted qualities such as literacy and a keen interest in acquiring knowledge [29]. Kim et al.‘s investigation on progress goals, learning strategies, and motivation in medical students found a correlation between academic goals, learning strategies, and motivation [30], aligning with the results of the present study. Universities of medical sciences, including dentistry, aim to nurture dedicated and skilled graduates responsive to societal health needs, necessitating consideration of students’ motivations in university planning.

Achievement motivation is the inclination to critically assess one’s own performance against the highest standards, actively pursue success, and derive pleasure from achieving performance milestones. These individuals possess the requisite motivation to effectively accomplish tasks, attain goals, and persist in their efforts until achieving a certain level of competence in their endeavors.

### Emotional coping

Students noted that stress diminishes their enjoyment of activities and hampers their learning. Liu et al.‘s study on Chinese high school students similarly indicated that classroom academic stress negatively impacts intrinsic motivation, which is consistent with the results of the present study. Reducing academic stress can increase students’ internal motivation and reduce their demotivation [31].

### Self-control

A high volume of courses and an intensive study program were raised, which is consistent with Ravipour et al.‘s findings [23]. Standardized educational planning and breaks between academic semesters could potentially enhance student motivation.

The need to work during studies reduces study time, echoing Banerjee’s research linking poverty, economic deprivation, and family issues to decreased academic motivation [32]. Cultural differences, highlighted in studies by Wentzel and Skinner, underscore the impact of family support on academic outcomes. In Iranian society, where family plays a central role, being away from family or family disputes can significantly influence students’ academic motivation. Most of the studies that have focused on the role of family problems in academic motivation have been on children and adolescents and have examined the role of family upbringing. However, in the present study, most cases related to this subclass were about being away from family or the role of family problems in married students. Also, in Western societies, the position of the family is different from Iran. Wentzel and Skinner’s study showed that cultural differences greatly impact the family’s role in academic performance. In societies with family support and bonding through cultural values and strong intra-family relationships, the family may be effective on academic outcomes and less adjustment problems [33].

### Situational perception

The large number of students in the theoretical classes and the low ratio of patients to students in the practical sections were among the issues raised in this research. Large classrooms pose challenges for effective teaching, often leading to isolation, reduced motivation, poor participation, lower attendance, and increased distractions like side conversations and mobile phone use [34].

### Environment

The concept of the environment was pivotal for students, encompassing social and physical environment categories. The university’s motivational and supportive atmosphere featured extracurricular activities, research encouragement, peer influence, patients’ inappropriate behavior, and a competitive student atmosphere.

According to participants, extracurricular activities and research positively impact academic motivation, citing examples like camping and research workshops. Research activities promote self-directed learning and expose students to various scientific fields, influencing academic motivation. Shojaei et al.‘s study indicated that sports involvement, even at the championship level, doesn’t hinder academic status in medical sciences universities, with a positive though statistically insignificant relationship [35]. Lumley et al.‘s research at Birmingham Medical School demonstrated better academic results for individuals engaged in research as a side activity [36]. However, Fares et al.‘s study suggested that excessive involvement in research and extracurricular activities could lead to increased stress and depression [37]. Hence, achieving a balance among academic commitments, rest, and extracurricular activities is crucial for students’ academic and psychological well-being.

Patients form a fundamental aspect of clinical education, and their cooperation is indispensable for effective learning. Challenges and inappropriate patient behaviors can impede clinical education and reduce student motivation. Marwan et al.‘s study emphasizes that patients generally view the presence of students positively during the treatment process [38]. Teaching communication skills and clarifying the student’s role to patients is essential to prevent inappropriate encounters.

The study participants suggest that fostering competition among students can boost motivation, with research indicating a strong correlation between competitiveness and grades [10]. However, for positive outcomes, this competition should align with scientific literacy. Merely pursuing high grades at the expense of collaboration can have detrimental effects, especially for dentists needing skills for effective teamwork within the health team.

Deficiencies in university service quality included dormitory facilities, educational resources, and university amenities. Zamani and Pouratashi’s findings align with our results, indicating a positive relationship between the educational environment and quality of infrastructures with motivation to progress [39]. Additionally, Rouhi et al.‘s study at Golestan University of Medical Sciences emphasizes the significance of welfare factors in education, highlighting the need to improve welfare and educational services [40]. This issue highlights the need to improve the quality of welfare and educational services.

The behavior of professors influences students and their learning. Professors’ behavior significantly impacts student motivation in clinical fields like dentistry, where the professor-student relationship is closer. As shown by Ramezani et al.‘s study, proper interaction effectively increases academic motivation [41].

Professors’ role in academic motivation is crucial, and their suitable teaching methods positively affect motivation [42]. The teaching quality was identified as a motivation influencer, consistent with Bavarsad et al.‘s findings [43]. However, Ramezani’s study showed no significant relationship between motivation and educational affairs [41], potentially influenced by differences in participants’ fields of study.

Tests are part of the learning process, and conducting tests is one of the key skills of the teaching profession [44, 45]. Therefore, the appropriateness and compatibility of evaluation methods with special learning goals should be ensured. Deficiencies in exam results clarity, stemming from discrepancies between self-evaluation and grades, pose a challenge, as well as the small difference between the grades of a student who has made a lot of effort and a student who has studied little. Ensuring evaluation methods align with learning goals is crucial.

The study identifies environmental factors, aligning with social cognitive theory, as key contributors to students’ academic motivation. Strengths of our study include the researcher’s familiarity with all Tabriz Dental Faculty students and the facilitation of diverse participant selection.

### Limitations

The results of this study show the views of a number of dental students in Tabriz. In addition, in this study, the type of university and its specific provincial and geographical location limits the generalization of the results. However, it may have something in common with other universities or even other fields. Participants’ reluctance to express true motivations due to consequences is acknowledged; for this purpose, at the beginning of each interview, the participants were assured that their names would not be recorded in any way and the results would only be reported in the form of code.

It is suggested that quantitative research be conducted to examine the relationship between the concepts resulting from this research and determine the contribution of each component to academic motivation.

## Conclusion

The views of Tabriz dental students regarding academic motivation were classified into seven themes: self-efficacy, outcome expectations, outcome expectancies, emotional coping, self-control, situational perception, and environment. This study’s findings can help find more effective interventions to improve the motivation level of dental students.

## Data Availability

The datasets used and analysed during the current study are available from the corresponding author on reasonable request.

## References

[1] Dweck CS, Dixon ML, Gross JJ. What Is Motivation, Where Does It Come from, and How Does It Work? In: Motivation Science. Oxford University Press. New York; 2023. p. 5-C1.1P23.

[2] Fardanesh H, Ebrahimzade I, Sarmadi M, Rezaie M, Omrani S (2012). A comparative study of Learning and Motivation in Continuing Medical Education based on Integrated Instructional and Motivational Design models. Iran J Med Sci.

[3] Saravani S, Mirzahosseini H, Zargham Hajebi M (2018). Explaining the Structural Model of Interactive relationships of Keller Motivational Dimensions with Personality Characteristics of Medical Students of Iran University of Medical Sciences. Razi J Med Sci.

[4] Gottfried AE (1990). Academic intrinsic motivation in Young Elementary School Children. J Educ Psychol.

[5] Sivrikaya AH (2019). The relationship between academic motivation and academic achievement of the students. Asia J Educ Train.

[6] Keller JM (2016). Motivation, Learning, and technology: applying the ARCS-V motivation model. Particip Educ Res.

[7] Javadi A, Faryabi R (2016). Relationship between motivation and academic performance in students at Birjand University of Medical Sciences. Educ Strategy Med Sci.

[8] Keller JM (2008). First principles of motivation to learn and e3-learning. Distance Educ.

[9] Bureau JS, Howard JL, Chong JXY, Guay F (2022). Pathways to Student Motivation: a Meta-Analysis of antecedents of Autonomous and controlled motivations. Rev Educ Res.

[10] Firouznia S, Yousefi A, Ghassemi G (2009). The relationship between academic motivation and academic achievement in Medical students of Isfahan University of Medical Sciences. Iran J Med Sci.

[11] Saeedi M, Parvizy S (2019). Strategies to promote academic motivation in nursing students: a qualitative study. J Educ Health Promot.

[12] Wasityastuti W, Susani YP, Prabandari YS, Rahayu GR (2018). Correlation between academic motivation and professional identity in medical students in the Faculty of Medicine of the Universitas Gadjah Mada Indonesia. Educación Médica.

[13] Amrai K, Motlagh SE, Zalani HA, Parhon H (2011). The relationship between academic motivation and academic achievement students. Procedia Soc Behav Sci.

[14] Bengtsson M, Ohlsson B (2010). The nursing and medical students motivation to attain knowledge. Nurse Educ Today.

[15] Squires A, Dorsen C (2018). Qualitative research in nursing and health professions regulation. J Nurs Regul.

[16] Hsieh HF, Shannon SE (2005). Three approaches to qualitative content analysis. Qual Health Res.

[17] Graneheim UH, Lundman B (2004). Qualitative content analysis in nursing research: concepts, procedures and measures to achieve trustworthiness. Nurse Educ Today.

[18] Stuckey H (2015). The second step in data analysis: coding qualitative research data. J Soc Health Diabetes.

[19] Jiang Y, Rosenzweig EQ, Gaspard H. An expectancy-value-cost approach in predicting adolescent students’ academic motivation and achievement. Contemp Educ Psychol. 2018;54.

[20] Yarin AJ, Encalada IA, Elias JW, Surichaqui AA, Sulca RE, Pozo F. Relationship between Motivation and Academic Performance in Peruvian Undergraduate Students in the Subject Mathematics. Palou E, editor. Educ Res Int. 2022; 2022: 3667076.

[21] Lam KKL, Zhou M. Grit and academic achievement: a comparative cross-cultural meta-analysis. J Educ Psychol. 2022;114.

[22] Schunk DH, DiBenedetto MK (2020). Motivation and social cognitive theory. Contemp Educ Psychol.

[23] Ravanipour M, Ranjbar Vakilabadi D (2019). Factors affecting Educational Motivation from the viewpoint of Environmental Health Engineering students: a content analysis. Med educ dev.

[24] Weiner B (1985). An attributional theory of achievement motivation and emotion. Psychol Rev.

[25] Buunk AP, Gibbons FX (2007). Social comparison: the end of a theory and the emergence of a field. Organ Behav Hum Decis Process.

[26] Dikici MF, Yaris F, Topsever P, Tuncay Muge F, Gurel FS, Cubukcu M, Gorpelioglu S (2008). Factors affecting choice of specialty among first-year medical students of four universities in different regions of Turkey. Croat Med J.

[27] Nedjat S, Emami Razavi H, Rashidian A, Yazdani S, Majdzadeh R (2006). The motives of medical students in Tehran University for Choosing Medicine Field and their outlooks for their Profession: qualitative versus quantitative Approach. Stride dev med educ.

[28] Neamatollahi H, Mehrabkhani M, Ghafarpour S, Ghasemi A (2014). Evaluation of Mashhad Dental School Students’ motives and viewpoints on their Career choices in 2010. J Mashhad Dent Sch.

[29] Pajares F (2003). Self-efficacy beliefs, motivation, and achievement in writing: a review of the literature. Read Writ Q.

[30] Kim S, Hur Y, Park JH (2014). The correlation between achievement goals, learning strategies, and motivation in medical students. J Med Educ.

[31] Liu Y (2015). The longitudinal relationship between Chinese high school students’ academic stress and academic motivation. Learn Individ Differ.

[32] Banerjee PA (2016). A systematic review of factors linked to poor academic performance of disadvantaged students in science and maths in schools. Cogent Educ.

[33] Wentzel K, Skinner E (2022). The other half of the story: the role of Social relationships and Social contexts in the development of academic motivation. Educ Psychol Rev.

[34] Nhokwara TB, Ashipala DO, Joel MH (2022). Lived experiences of nursing students regarding learning in large classes and its effects on teaching and learning at the University of Namibia. Curationis.

[35] Shojaee M, Ranjbar A, Sharifian E (2010). Investigating the relationship between academic and sports indicators of sports elites of medical sciences universities in the country. J Sport Manag Motor Behav.

[36] Lumley S, Ward P, Roberts L, Mann JP (2015). Self-reported extracurricular activity, academic success, and quality of life in UK medical students. Int J Med Educ.

[37] Fares J, Saadeddin Z, Al Tabosh H, Aridi H, El Mouhayyar C, Koleilat MK (2016). Extracurricular activities associated with stress and burnout in preclinical medical students. J Epidemiol Glob Health.

[38] Marwan Y, Al-Saddique M, Hassan A, Karim J, Al-Saleh M (2012). Are medical students accepted by patients in teaching hospitals?. Med Educ Online.

[39] Naseh L, Mardanian Dehkordi L, Naseh H. Investigation of Educational Motivation and Its Related Factors in Students of Shahrekord University of Medical Sciences. JMED. 2017;12(1 and 2):27–39.

[40] Esmaeili MR, Hozni SA, Mosazadeh B, Zavareh A (2017). Good teacher’s characteristics and its influence on Dental Students Academic Motivation in Guilan University of Medical Sciences. Res Med Educ.

[41] Ramezani G, Pourbairamian G, Aalaa M, Moradi E, Habibi H, Bigdeli S. Educational Motivation Improvement Strategies for Postgraduate Students: Nominal Group Technique. DSME 2021;8(1):1–12.

[42] Maulana R, Opdenakker MC, Bosker R (2016). Teachers’ instructional behaviors as important predictors of academic motivation: changes and links across the school year. Learn Individ Differ.

[43] Bakhshandeh bavarsad M, Hakim AS, Azimi N, Latifi M, Ghalvandi H (2015). Nursing students viewpoints about Educational Motivation and its related factors in Ahvaz Jundishapur University of Medical Sciences. Res Med Educ.

[44] Ramazani S, Ramazani H, Ramazani A, Hemmati M (2020). Investigation of Educational Motivation and its related factors of the students of Laboratory sciences of the Eastern Medical sciences universities. J Med Educ Dev.

[45] Black P, Wiliam D (2009). Developing the theory of formative assessment. Educ Assess Eval Acc.

